# Ratio of observed to predicted deaths in pediatric patients after introducing a closing policy in a general ICU

**DOI:** 10.1186/cc9912

**Published:** 2011-03-11

**Authors:** Y Ueno, H Imanaka, J Oto, M Nishimura

**Affiliations:** 1 The University of Tokushima Graduate School, Tokushima, Japan; 2 Tokushima University Hospital, Tokushima, Japan

## Introduction

The purpose of this study was to investigate whether the introduction of a closed ICU policy affected the prognosis of the critically ill pediatric patients in a general ICU.

## Methods

Our ICU is a general acute-care one. The Department of Emergency and Critical Care Medicine was established in January 2004. Since then, full-time intensivists performed daily rounds and decided the ventilatory setting, cardiovascular treatment and antimicrobial agents (closed policy). We collected the Pediatric Index of Mortality 2 (PIM2) score for each pediatric patient (≤15 years old) admitted to our ICU from 2001 to 2009. We divided the patients into three terms: the early (2001 to 2003), middle (2004 to 2006), and latest (2007 to 2009) groups. We obtained the predicted number of deaths by summing the PIM2 score for every patient. We compared the ratio of observed to predicted deaths (O/P ratio) between the three groups.

## Results

The patient profile and results are shown in Tables 1 and 2. In total, 532 pediatric patients were collected. The PIM2 score increased significantly from 0.066 ± 0.130 in 2001 to 2003 to 0.114 ± 0.239 in 2004 to 2006 and to 0.086 ± 0.147 in 2007 to 2009. However, the O/P ratio decreased from 1.49 in 2001 to 2003 to 0.82 in 2004 to 2006 and 0.82 in 2007 to 2009.

**Table 1:** Patient profile

	2001 to 2003	2004 to 2006	2007 to 2009
Total	194	181	157
Male	90 (46%)	94 (52%)	67 (43%)
Age (years)	2.8 ± 3.7	3.5 ± 3.9	4.1 ± 4.6

**Table 2:** Results

	2001 to 2003	2004 to 2006	2007 to 2009
PIM2 score	0.066 ± 0.130	0.114 ± 0.239	0.086 ± 0.147
Observed death	19	17	11
Sum of PIM2	12.75	20.71	13.43
O/P ratio	1.49	0.82	0.82

## Conclusions

The O/P ratio improved after the establishment of a closed policy in our general ICU.
